# Methods for the Induction of Reproduction in a Tropical Species of Filamentous *Ulva*

**DOI:** 10.1371/journal.pone.0097396

**Published:** 2014-05-13

**Authors:** Christina Carl, Rocky de Nys, Rebecca J. Lawton, Nicholas A. Paul

**Affiliations:** MACRO – the Centre for Macroalgal Resources and Biotechnology, and School of Marine and Tropical Biology, James Cook University, Townsville, Queensland, Australia; Auckland University of Technology, New Zealand

## Abstract

The green seaweed *Ulva* is a major fouling organism but also an edible aquaculture product in Asia. This study quantified for the first time the effect of key factors on the reproduction of a tropical species of filamentous *Ulva* (*Ulva* sp. 3). The controlled timing of release of swarmers (motile reproductive bodies) was achieved when experiments were initiated in the early afternoon by exposing the thalli to a temperature shock (4°C) for 10 min and subsequently placing them into autoclaved filtered seawater under a 12 h light: 12 h dark photoperiod at 25°C. The release of swarmers then peaked two days after initiation. In contrast, segmentation, dehydration, salinity or time of initiation of experiments had no effect of any magnitude on reproduction. The released swarmers were predominantly biflagellate (95%), negatively phototactic and germinated without complementary gametes. This indicates that *Ulva* sp. 3 has a simple asexual life history dominated by biflagellate zoids.

## Introduction

The green seaweed *Ulva* (syn. *Enteromorpha*; [1]) is a major fouling organism on marine infrastructure [2]–[4], with the prevention of settlement and growth of this genus being a primary goal for antifouling technologies [5]–[7]. Paradoxically, *Ulva* is also an important high-value aquaculture product in Japanese and Asian cuisine, known as ‘aonori’ [8] with economic benefits relying on the enhanced settlement and growth of these species [9]–[12]. Finally, *Ulva* is a target for the bioremediation of nitrogen and phosphorous from aquaculture with a similar goal to aquaculture of enhanced growth [13]–[16].

The key to developing both antifouling and aquaculture technologies for *Ulva* is the manipulation of reproduction thereby inducing the release of swarmers to be used as bioassay test organisms or as seedlings. The term ‘swarmer’ refers to motile reproductive bodies of *Ulva*, the nature of which depends on the three life history stages from which the swarmers are sourced. This can be either sexual or one of two asexual life history stages [17]. The sexual life history is characterised by an alternation of isomorphic sporophytes and gametophytes [18], with both generations only being distinguishable by their reproductive structures [19], [20]. Sporophytes release quadriflagellate zoospores, whereas gametophytes release biflagellate female (+) or male (−) gametes which fuse and form zygotes. In contrast, *Ulva* species possessing a simple asexual life history produce either exclusively biflagellate or quadriflagellate zoids [17], [21]. In all cases, pre-existing vegetative cells of *Ulva* transform directly into reproductive cells [19], [22].

The liberation of zoospores and gametes is driven by lunar periodicity in temperate and cold waters [10], [12], [23]–[25]. In addition, the formation and release of zoospores, gametes, and asexual zoids of *Ulva* in temperate environments is induced by a range of stimuli including dehydration [26], [27], extended dark periods over several days [27], [28], fragmentation [12], [29]–[33], renewal of culture medium [28], [34], salinity [12], and change in temperature [29], [30], [35], [36]. In contrast, the identified stimuli that induce the reproductive development of *Ulva* in tropical environments are restricted to salinity, dehydration, segmentation, and temperature [37]–[40], and there is a lack of understanding of other key drivers controlling reproduction, particularly for filamentous species of *Ulva* (formerly *Enteromorpha*).

Given the economic importance of *Ulva* and its capacity for rapid and intense growth under tropical conditions [15], [41], [42], there is an imperative to understand the mechanisms that influence the reproduction of this species in tropical environments. Therefore, the aim of this study was to understand the fundamental physiological drivers of reproduction in the most common filamentous species of tropical *Ulva* (*Ulva* sp. 3) in Eastern Australia [42], and subsequently develop manipulative treatments to control reproduction. This will provide a baseline method for the reliable supply of swarmers with a direct application to laboratory fouling studies and the commercial production of filamentous *Ulva* in the tropics. The effects of six key factors on reproduction of *Ulva* sp. 3 were quantified in laboratory-based experiments. These were (1) salinity, (2) dehydration, (3) segmentation, (4) photoperiod, (5) time of initiation of experiments, and (6) temperature shock. Furthermore, the most successful combination of treatments was used to quantify the reproductive output of *Ulva* sp. 3, the number of flagella and phototactic response of the swarmers and their ability to settle and germinate.

## Materials and Methods

### Study Species and Collection

**Figure 1:**
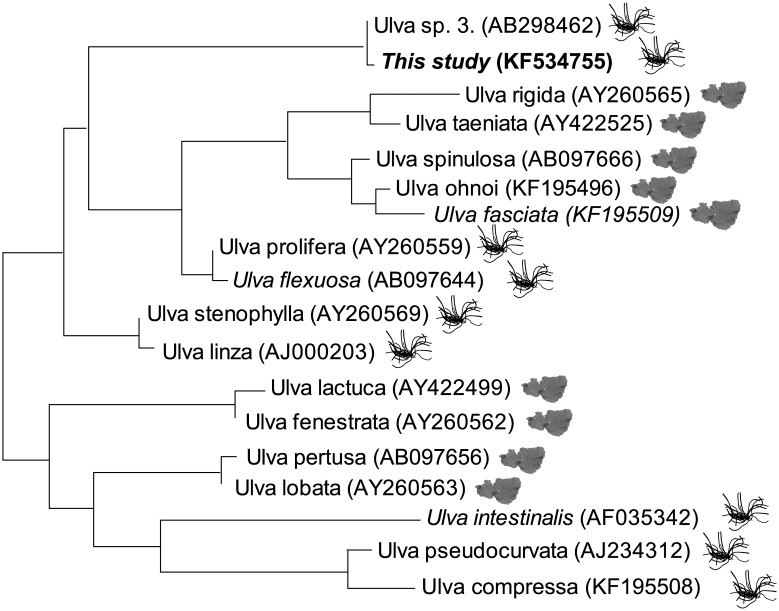
Reduced *Ulva* ITS phylogenetic tree. Reduced version of maximum likelihood tree of *Ulva* ITS sequences (Figure S1), showing the species used in this study (shown in bold) and *Ulva* species used in previous studies investigating the reproduction of this genus. Species investigated in tropical environments are shown in italic. Pictures next to species represent the morphology (flat and blade-like or tubular and filamentous thalli).

The genus *Ulva* has a worldwide distribution with a broad tolerance of salinity, with species occurring in hypersaline to freshwater environments [43]–[45]. The species used in this study is characterised by flat tubular thalli. Because of the broad intraspecific variation and taxonomic difficulties associated with the *Ulva* genus [46], the species used in this study was analysed using molecular barcoding. Barcoding compares short DNA sequences from a standardised region of the genome - the ‘barcode’ - to a library of reference sequences derived from individuals of known identity [47]. The species was identified as *Ulva* sp. 3 [43] (see also Text S1) using newly generated DNA sequences from the internal transcribed spacer (ITS) region of the ribosomal cistron (Genbank accession number KF534755). Phylogenetic trees constructed using these sequences show that this species occupies a unique clade that is distinct from all other filamentous species of *Ulva* used in previous studies of reproduction (Figure 1; see Figure S1).

*Ulva* sp. 3 (hereafter *Ulva*) was collected by hand from a land-based aquaculture facility at Guthalungra (19° 55′S, 147° 50′E), Queensland, Australia. Permission was obtained from owners to collect algae from this site. Notably, reproductive patterns driven by lunar periodicity as found for temperate species of *Ulva* [25], [48] were not observed in the field populations collected over a four month period. Samples were placed in a 25 L container filled with pond water and then transported within 3 h to the laboratory at James Cook University (JCU) in Townsville, Australia. Subsequently, the samples were gently washed three times with filtered seawater (FSW; 0.2 µm and UV sterilised) to remove debris, epiphytes and invertebrates.

### Laboratory Experiments

#### Effect of salinity, dehydration, and segmentation

To determine the effect of salinity, dehydration and segmentation on the formation and release of swarmers, these factors were manipulated in a fully factorial experiment under laboratory conditions using samples collected in the morning and gently cleaned with FSW as described above. Firstly, *Ulva* was exposed to a lower salinity by placing filaments in dechlorinated tap water (DC) for 10 min with FSW being used as a salinity control. Secondly, the effect of dehydration was tested for each salinity treatment. All filaments were dried using paper towels to remove excess water and subsequently either dehydrated by exposure to air for 45 min in the dark at 25°C (dehydration treatments), or alternatively placed in FSW in the dark for 45 min (non-dehydrated control). Thirdly, the effect of segmentation was tested by cutting a section of approximately 50–70 mm from each filament (hereafter referred to as ‘whole’), or alternatively cutting this section further into pieces <5 mm using a razor blade (hereafter referred to as ‘cut’). Both treatments had similar biomass per unit volume. Each of the whole and cut filaments were placed in individual Petri dishes (Iwaki; 1010-060) filled with 10 mL autoclaved FSW and sealed using Parafilm to prevent evaporation. The Petri dishes were then placed in a culture cabinet at 25°C at an irradiance of 125 µmol photon m^−2^ s^−1^ under a 12 h light (L)∶12 h dark (D) photoperiod for three days. A total of three replicates was used for each treatment combination (*n* = 3 for each salinity×dehydration×segmentation).

**Figure 2:**
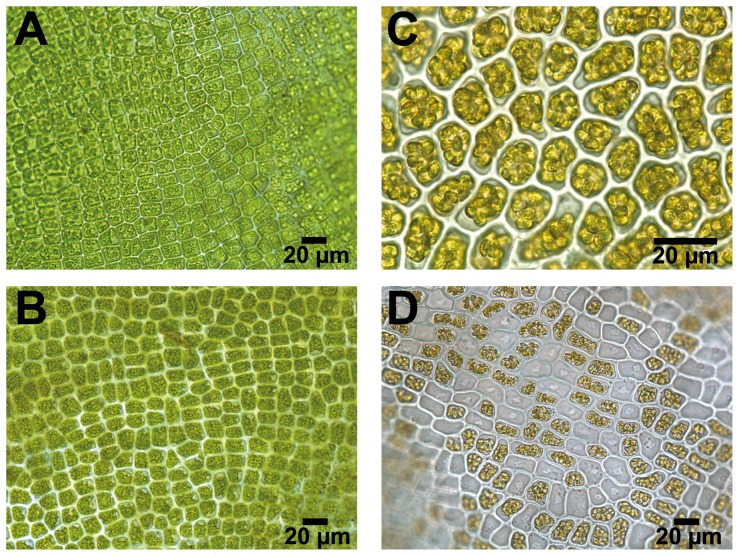
Light micrographs of *Ulva* sp. 3. Transformation of vegetative cells into reproductive cells of *Ulva* sp. 3. (**a**) Vegetative cells, (**b**) formation of swarmers after 26 h, (**c**) reproductive cells with fully formed swarmers after 44 h, (**d**) reproductive cells with fully formed and discharged swarmers after 46 h. Scale bar, 20 µm.

The percentage of the total area of each filament (either whole or cut) that had released swarmers (hereafter referred to as ‘discharge’) was visually quantified daily at 3 pm using a dissecting microscope (Olympus SZ61) according to Nielsen and Nordby [49]. Vegetative cells of *Ulva* transform directly into reproductive cells (Figure 2). The vegetative (Figure 2a), reproductive (Figure 2c) and discharged cells (Figure 2d) had a green, brown-ish and white colour, respectively [19], [50]. The discharge was quantified using only white cells that had unequivocally released swarmers (Figure 2d).

#### Effect of time of initiation of experiments and photoperiod

To determine the effect of time of initiation of experiments and photoperiod, on the formation and release of swarmers, samples were collected in the morning and gently cleaned with FSW as described above. Algae were subsequently maintained in an outdoor aquarium at the Marine & Aquaculture Research Facilities Unit at JCU until experiments were initiated by cutting a single section from each filament. Experiments were initiated at five times, two on the day of collection at 1 pm and 7 pm, and three on the day after collection at 7 am, 1 pm and 7 pm. The times of initiation affected the length of exposure to light and darkness at the start of the experiment (experiments initiated at 7 am were initially exposed to 12 h light in contrast to experiments initiated at 7 pm where thalli were initially exposed to 12 h darkness).

For all replicates, a single section of approximately 50–70 mm was cut from each filament (see Results) and placed in Petri dishes (Techno Plas; S6014S10) filled with 10 mL autoclaved FSW and sealed with Parafilm. A total of 1080 replicate dishes were then placed in one of three culture cabinets at 25°C at photoperiods of a normal light period (12 h L∶12 h D), an extended light period (18 h L∶6 h D), or constant light (24 h L). In addition, another 360 Petri dishes were wrapped in aluminium foil to provide constant darkness (24 h D) and these were split between the culture cabinets.

The discharge of swarmers was quantified at 7 pm on the day of collection and then daily at 7 am, 1 pm, and 7 pm for the following three days. At each sampling point, nine haphazardly selected dishes were destructively sampled from each treatment (*n* = 9 for each photoperiod×time of experimental initiation) and the discharge quantified as a percentage of the total area of each filament, as described above.

#### Effect of temperature shock and controlled release of swarmers

This experiment determined the effect of temperature shock on the formation of swarmers and assessed the potential to control their release by initially constraining and subsequently inducing the immediate release of swarmers to rapidly obtain a dense suspension of swarmers. Samples were collected in the morning and gently cleaned with FSW as described above. In the early afternoon, filaments of *Ulva* were placed in chilled (4°C) FSW and stored in the fridge for 10 min prior to placing a single section of approximately 50–70 mm from each filament (see Results) in Petri dishes (Techno Plas; S6014S10) filled with 10 mL autoclaved FSW. The use of a 10-min period was based on a pilot experiment where extended exposure periods to 4°C did not increase the formation and release of swarmers. As controls, filaments were either placed in 25°C FSW and stored in the dark for 10 min or immediately used in assays after the cleaning procedure without any further pre-treatment. The dishes were then placed in a culture cabinet at 25°C at an irradiance of 125 µmol photon m^−2^ s^−1^ under a 12 h L∶12 h D photoperiod.

After two days in the culture cabinet, prior to the expected onset of the release of swarmers, filaments were wrapped in moist paper towels at 7 am or exposed to air for 4 h by placing each filament individually on baking paper. After 4 h (11 am), each filament was placed in a new Petri dish filled with 10 mL autoclaved FSW. These methods were used in an attempt to constrain the release of swarmers to a short period of time in order to rapidly obtain a dense suspension of swarmers. A control remained submersed over this same period of time. The time of day selected reflected the onset of release in previous experiments (see Results). The discharge of swarmers was determined at 11 am within 5 min post re-submersion, and at 4 pm. A total of ten replicates was used for each treatment combination (*n* = 10 for temperature pre-treatment×restraining-treatment).

#### Reproductive output

The reproductive output (the number of swarmers released per unit area) of *Ulva* was estimated by quantifying the surface area of individual thalli and subsequently determining the percentage area of released swarmers, and the numbers of discharged swarmers over time for each thallus. Samples of *Ulva* were collected in the morning and gently cleaned with FSW as previously described. The most successful combination of treatments tested above was used to induce sporulation and control the release of swarmers (see Results). Briefly, the samples were chilled at 4°C for 10 min to maximise the formation and release of swarmers prior to cutting a section of approximately 50–70 mm from filaments (*n* = 50). Images of the filament pieces were captured using a camera (Olympus DP25) attached to a dissecting microscope (Olympus SZ61) for surface area measurements (Image J). Subsequently, these single filaments were placed in Petri dishes filled with 10 mL autoclaved FSW, which were then placed in a culture cabinet at 25°C at an irradiance of 125 µmol photon m^−2 ^s^−1^ under a 12 h L∶12 h D photoperiod.

To determine the number of released swarmers over time, two days after the initiation of experiments each filament was transferred into a new Petri dish filled with 10 mL autoclaved FSW every 90 min (from 7 am until 1 pm). This time period was the window of peak release for swarmers (see Results). To obtain accurate numbers of discharged swarmers in the water column, the dishes and water were changed at each measurements point (every 90 min) to minimise settlement. The water in the dishes was preserved in 1% Lugol’s solution and subsequently transferred into sealable sample tubes. To increase the concentration of swarmers, the samples were centrifuged (400 *g* for 1 min; [51]) and concentrated to a volume of 2.5 mL. Subsequently, the number of swarmers was determined using a haemocytometer. The reproductive output (RO) of each filament was calculated using the equation *RO = NoS/(SA·D)*, where *NoS* is the number of released swarmers, *SA* the surface area of each filament and *D* is the discharge (percentage area of swarmer release). Finally, the number of flagella on released swarmers was counted using a compound microscope (Olympus BX53). The number of flagella on approximately 20 swarmers per sample was quantified in order to determine whether swarmers were bi- or quadriflagellate. Due to the low discharge of some thalli, only 59 samples could be used for counting the number of flagella on swarmers.

In addition, the viability and germling development of released swarmers was determined by inducing sporulation in a second section from each of the 50 filaments. These filaments were placed in individual Petri dishes filled with 10 mL autoclaved FSW and incubated as previously described to induce sporulation. After two days, the filaments were removed at 2 pm and nutrients were added to each dish (AlgaBoost *1000x* f/2). Subsequently, the dishes were returned to the culture cabinet at 25°C under a 12 h L:12 h D photoperiod. After a culture period of five days, the swarmers released by each filament were examined for germination using an inverted microscope (Olympus CKX41).

Experiments were conducted with three independent collections of *Ulva* over an eight day period with 50 replicates from each collection time.

#### Phototactic behaviour of released swarmers

To determine the phototactic behavior of released swarmers, sporulation was induced by chilling samples and subsequently incubating an excised section of filaments (*n* = 40) in individual Petri dishes as previously described. After two days, the dishes were placed on a window sill at noon and any lights in the laboratory were turned off so that natural light was the primary light source. Released swarmers showed phototactic responses and consequently concentrated on the dark side of the dish facing away from the natural light when negatively phototactic or on the side with natural light source when possessing a positive phototaxis. Phototactic swarmers were removed and collected in sealable sample tubes over 90 min using a transfer pipette and preserved in 1% Lugol’s solution. Subsequently, the number of flagella was counted using a microscope (Olympus BX53). In addition, images of a total of 64 released swarmers were taken for size measurements (width and length).

### Statistical Analysis

Data were analysed by permutational analysis of variance (PERMANOVA) using PRIMER 6 (v. 6.1.13) and PERMANOVA+ (v. 1.0.3) [52]. The Bray-Curtis dissimilarity measure was used for all PERMANOVAs and *p*-values were calculated using permutation of residuals under a reduced model with 9999 random permutations. If there was a significant difference, pair-wise *a posteriori* comparisons were made among the significant groups using the Bray-Curtis similarity measure (α = 0.05). All data are reported as mean±1 standard error (S.E.) unless stated otherwise.

The effects of salinity shock, dehydration, and segmentation on the discharge of swarmers were considered as fixed factors in the first experiment. Because the second experiment evaluated the interactive effects of different times of initiation and photoperiod, these data had to be analysed in two ways. A formal comparison using PERMANOVA was made between the effects of time of initiation and photoperiod by treating the day of sampling (day 3 and 4) and the sampling time of day (7 am, 1 pm, and 7 pm) as fixed factors because dishes were destructively sampled. An alternative plot of the effect of time of initiation (5 initiation times over 2 days past sampling) was then made for the key photoperiod (12 h L:12 h D, see Results) by standardising the time of initiation at 0 h but was not formally analysed. The third experiment assessing the effect of temperature shock and treatments to control the release of swarmers, PERMANOVAs were run for each sampling point (11 am and 4 pm) with pre-treatment and restraining-treatment as fixed factors. To test for the effect of batch on the release of swarmers over time in the fourth experiment, a two-factor PERMANOVA was used with time as a fixed factor and batch as a random factor.

## Results

### Effect of Salinity, Dehydration, and Segmentation

**Figure 3:**
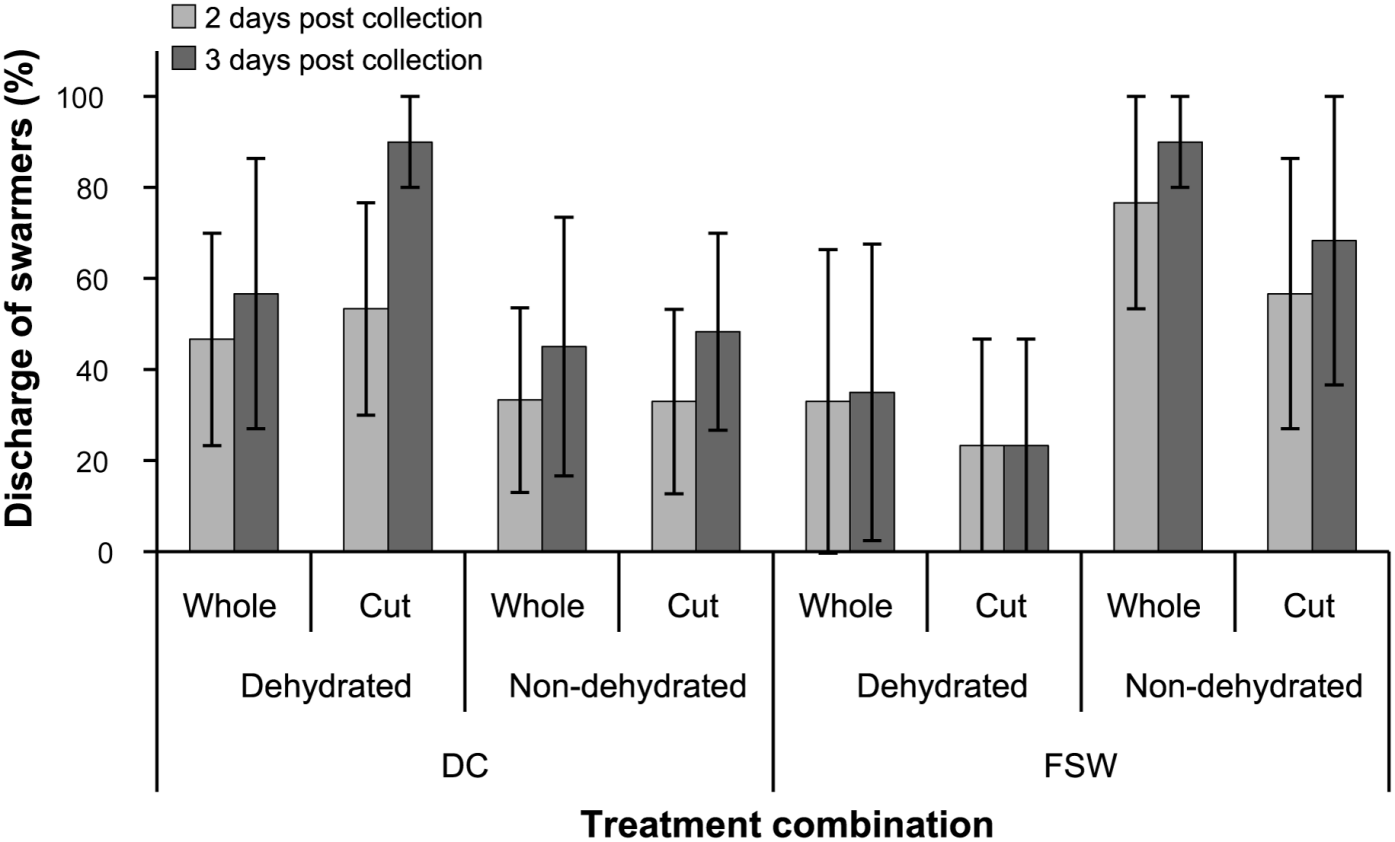
Discharge of swarmers in experiments testing the effect of salinity, dehydration, and segmentation. Mean (± S.E.) discharge of swarmers (%) after two and three days post collection and treatment. Samples were rinsed in dechlorinated tap water (DC) or filtered seawater (FSW) 10 min prior to dehydration for 45 min (Dehydrated). Non-dehydrated filaments (Non-dehydrated) were used as a control. Filaments were either left whole (Whole) or segmented into pieces <5 mm (Cut).

There was no effect of salinity shock (three-factor PERMANOVA: *F_(1, 16)_* = 0.86, *p* = 0.382), dehydration (*F_(1, 16)_* = 0.55, *p* = 0.512), or segmentation (*F_(1, 16)_* = 0.04, *p* = 0.948) on the discharge of swarmers among treatments after two and three days (Figure 3). No swarmers were released one day after initiation of experiments. After two days, the discharge of swarmers ranged from 23.3±23.3% (FSW×dehydrated×segmented) to 76.7±23.3% (FSW×non-dehydrated×whole) and was generally lower than after three days, where the discharge ranged from 23.3±23.3% (FSW×dehydrated×segmented) to 90.0±10.0% (FSW×non-dehydrated×whole, and also, DC×dehydrated×segmented). Therefore, the treatment combination of FSW×non-dehydrated×whole was chosen for the subsequent experiments on the basis of maximised discharge of swarmers with the least number of treatments.

### Effect of Time of Initiation of Experiments and Photoperiod

**Figure 4:**
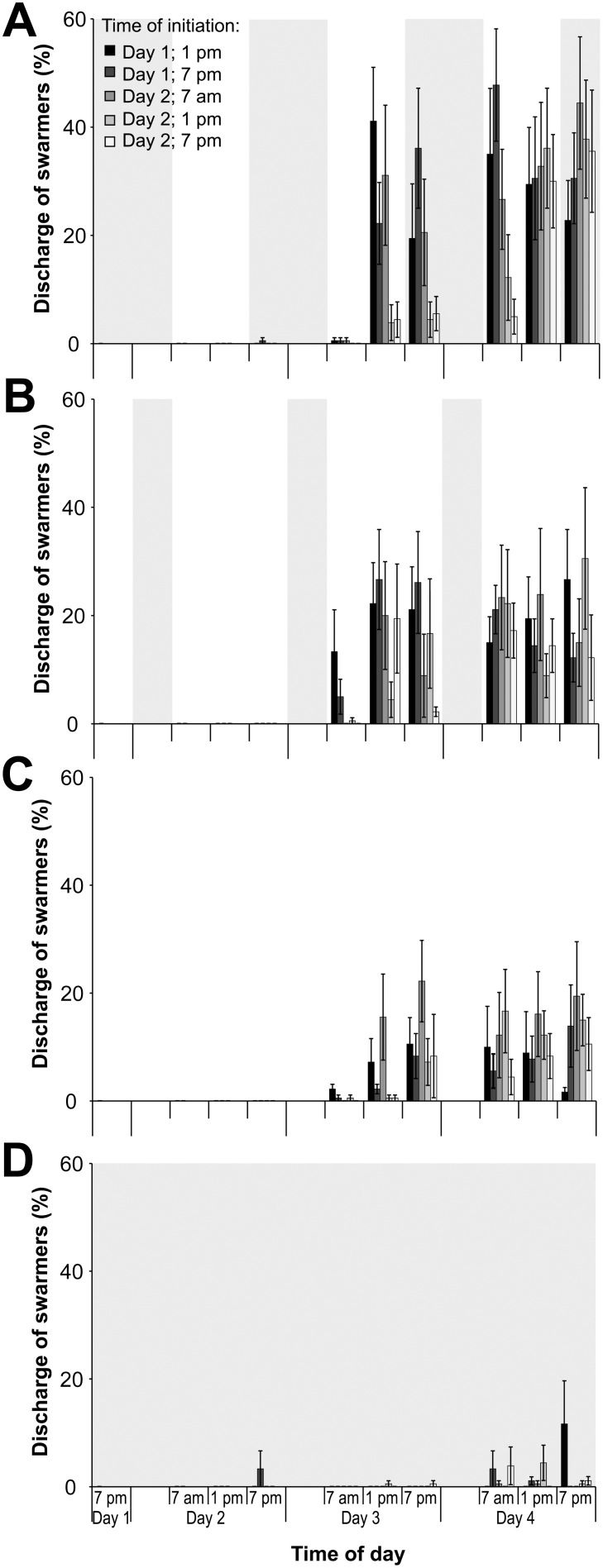
Discharge of swarmers testing the effect of time of initiation of experiments and photoperiod. Mean (± S.E.) discharge of swarmers (%) under photoperiods of (**a**) 12 h light:12 h dark; (**b**) 18 h light:6 h dark; (**c**) constant light; and (**d**) constant darkness. Experiments were initiated at 1 pm and 7 pm on the day of collection (Day 1) and at 7 am, 1 pm, and 7 pm one day after collection (Day 2). Grey shaded background indicates duration of the dark period.

The discharge of swarmers varied significantly between photoperiods (Figure 4), with the highest discharge in the 12 h L:12 h D photoperiod (47.8±10.4%). The discharge of swarmers decreased with extended light periods and was below 31% and 23% at photoperiods of 18 h L:6 h D and 24 L, respectively. The lowest overall discharge occurred under constant darkness, with less than 12% discharge at any time (Figure 4). Photoperiod had a significant interactive effect with time of initiation (*p*<0.001; Table 1), sampling day (*p*<0.001) and time of sampling day (*p* = 0.001). Furthermore, there was a complex interactive effect of photoperiod, sampling day, and time of sampling day on the discharge of swarmers (*p*<0.047; Table 1). This effect was driven by significant differences in the release of swarmers between photoperiods and higher overall discharge on day 4 in comparison to day 3.

**Table 1:** PERMANOVA output testing the effect of photoperiod (Ph; 12 h light:12 h dark, 18 h light:6 h dark, 24 h light, and 24 h dark), time of initiation (In; 1 pm and 7 pm on collection day, 7 am, 1 pm, and 7 pm one day after collection), sampling day (Day; 3 and 4 days past collection), and time of sampling day (sTime; 7 am, 1 pm, and 7 pm) (all fixed factors) on the discharge of swarmers.

Source	*df*	*F*	*P*
Ph	3	**68.05**	**<0.001**
In	4	**5.01**	**<0.001**
Day	1	**68.66**	**<0.001**
sTime	2	**12.89**	**<0.001**
Ph×In	12	**2.67**	**<0.001**
Ph×Day	3	**5.33**	**<0.001**
Ph×sTime	6	**2.81**	**0.001**
In×Day	4	1.72	0.101
In×sTime	8	0.90	0.549
Day×sTime	2	**6.83**	**<0.001**
Ph×In×sTime	12	1.08	0.356
Ph×In×sTime	24	0.89	0.688
Ph×Day×sTime	6	1.43	0.140
In×Day×sTime	8	**1.70**	**0.047**
Ph×In×Day×sTime	24	0.75	0.886
Residual	960		

Regardless of the time of initiation, the number of released swarmers were similar at the end of the experiment for each photoperiod and ranged from 22.8±7.4% to 44.4±12.2% under the normal photoperiod (12 h L:12 h D), with generally large variations in the discharge between samples of the same treatment combinations.

**Figure 5:**
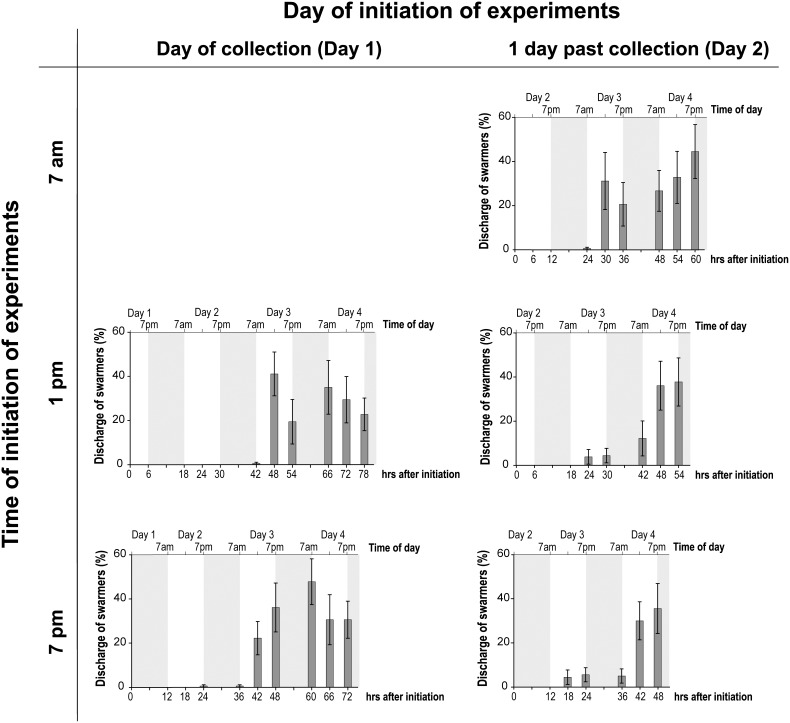
Discharge of swarmers under a 12;12 h D photoperiod testing the effect of time of initiation of experiments. Mean (± S.E.) discharge of swarmers (%) under a 12 h light:12 h dark photoperiod. Experiments were initiated at 1 pm and 7 pm on the day of collection (Day 1), and at 7 am, 1 pm, and 7 pm one day after collection (Day 2). Grey shaded background indicates the dark period.

Under the normal photoperiod (12 h L:12 h D), the discharge generally peaked between 42 and 48 h after the initiation of experiments (two days post initiation), with the exception of the early initiation of 7 am one day past collection where the discharge peaked after only 30 h (Figure 5). However, the same trend of an onset of release in the morning after the filaments were exposed to light occurred under normal photoperiod across all initiation times.

### Effect of Temperature Shock and Controlled Release of Swarmers

**Figure 6:**
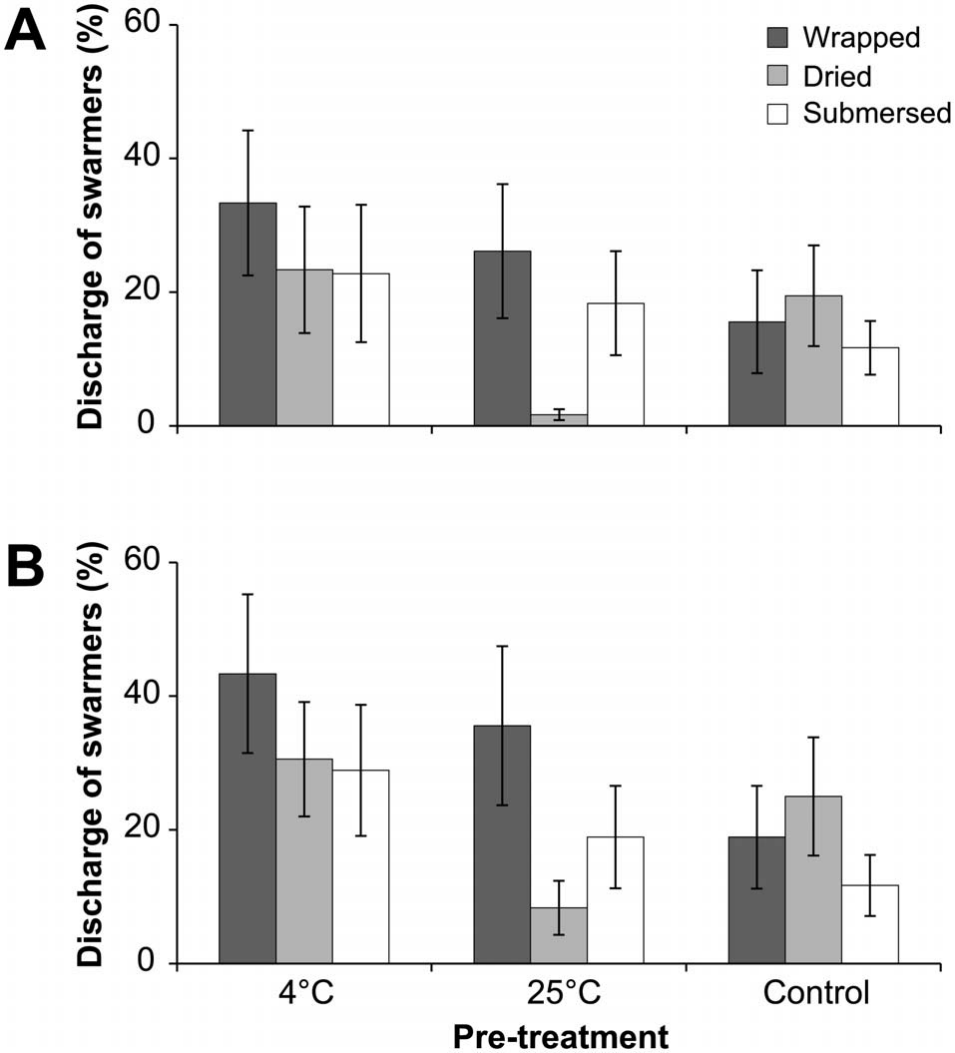
Discharge of swarmers testing the effect of temperature shock and controlled release of swarmers. Mean (± S.E.) discharge of swarmers (%) two days after initiation of experiments at (**a**) 11 am and (**b**) 4 pm. Filaments of *Ulva* were exposed to 4°C and 25°C FSW and without pre-treatment as a control. After two days, the filaments were wrapped in moist paper, dried, or remained submersed from 7 am for 4 h.

In general, the discharge of swarmers was higher for chilled filaments (4°C) than the other treatments (Figure 6), with the mean discharge being nearly double (34.4±4.6%) that of both the 25°C pre-treatment (20.9±7.9%) and without any pre-treatment (18.5±3.9%). However, the variance within the pre-treatments was relatively high (Figure 6) and pre-treatment was not a significant effect (two-factor PERMANOVA: *F*_(2,81)_ = 1.88, *p* = 0.107). There was no clear effect of restraining-treatment combinations to manipulate the release of swarmers (*F*_(2,81)_ = 0.57, *p* = 0.718).

The discharge of swarmers was not constrained for filaments wrapped in moist paper towel or dried for 4 h (Figure 6a). In fact, treatments to constrain the discharge of swarmers resulted in slightly higher discharge at 11 am than continuously submersed filaments, with the exception of the 25°C pre-treatment (submersed: 18.3±7.8%; dried; 1.7±0.8%; wrapped: 26.1±10.0%). The unwrapping of filaments at 11 am revealed a high discharge of swarmers on the moist paper towel and these were clearly visible due to the change from white to green/brown-ish colour of the paper towel. Furthermore, constraining the discharge of swarmers by wrapping and drying was unsuccessful indicated by similar discharge between 11 am and 4 pm for those filaments (Figure 6a, b). Up to 33% of the biomass discharged swarmers while being wrapped (Figure 6a), whereas the discharge ranged from 18.9±7.7% (control) to 43.3±11.9% (4°C) after being re-submersed for 5 h (4 pm; Figure 6b). Similarly, the discharge of dried filaments was up to 23% while exposed to air (Figure 6a) and increased marginally at 4 pm, ranging from 8.3±4.0 (25°C) to 30.6±8.5% (4°C) (Figure 6b).

### Reproductive Output

**Figure 7:**
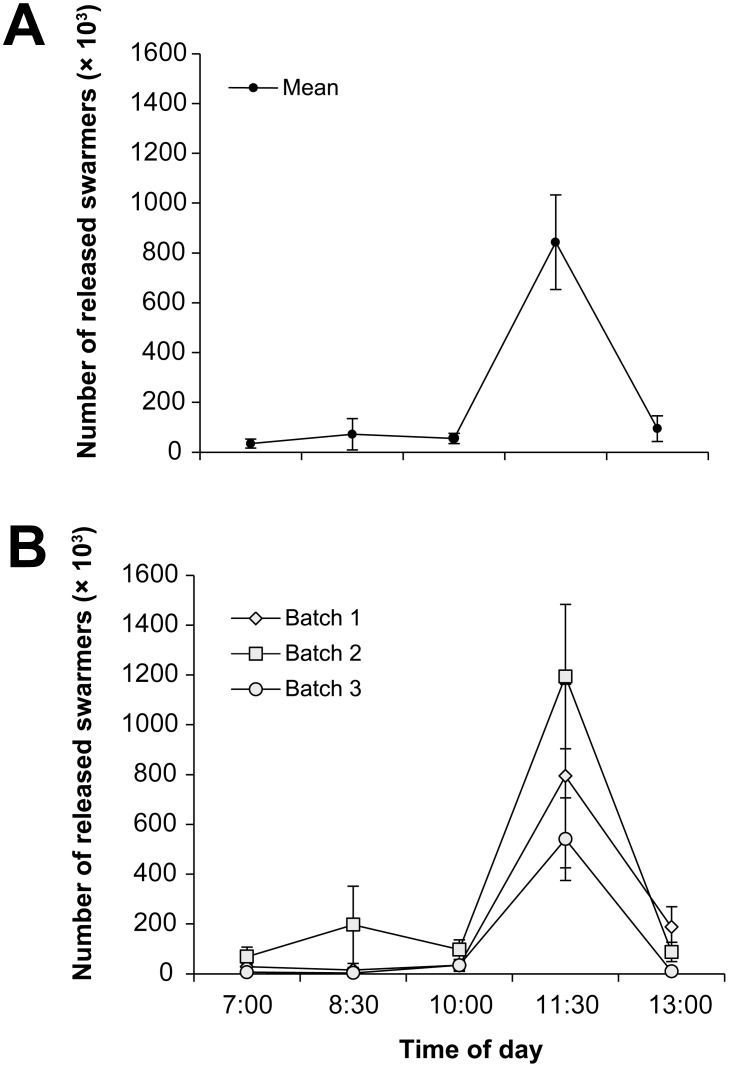
Number of released swarmers over time. (**a**) Mean (± S.E.) number of released swarmers over time (*n = *3). (**b**) Mean number (± S.E.) of released swarmers over time from three independently collected batches of algal biomass (*n = *50). Algal batches were collected on 6 May 2013 (Batch 1), 7 May 2013 (Batch 2), and 14 May 2013 (Batch 3).

The time of day had a significant effect on the number of swarmers released (two-factor PERMANOVA: *F_(4, 735)_* = 14.19, *p* = 0.003), with a peak of release at 11∶30 am with 842,708±190,123 swarmers released (mean of three batches ±1 SE) (Figure 7a). The number of swarmers released was an order of a magnitude smaller at all other times and ranged from 34,375±18,311 (7 am) to 94,791±51,549 (1 pm). There was also a significant effect of batch on the number of swarmers released (*F_(2, 735)_* = 3.16, *p* = 0.025); however, the number of swarmers released showed a similar trend among batches and the highest release consistently occurred at 11∶30 am, regardless of batch (Figure 7b).

**Figure 8:**
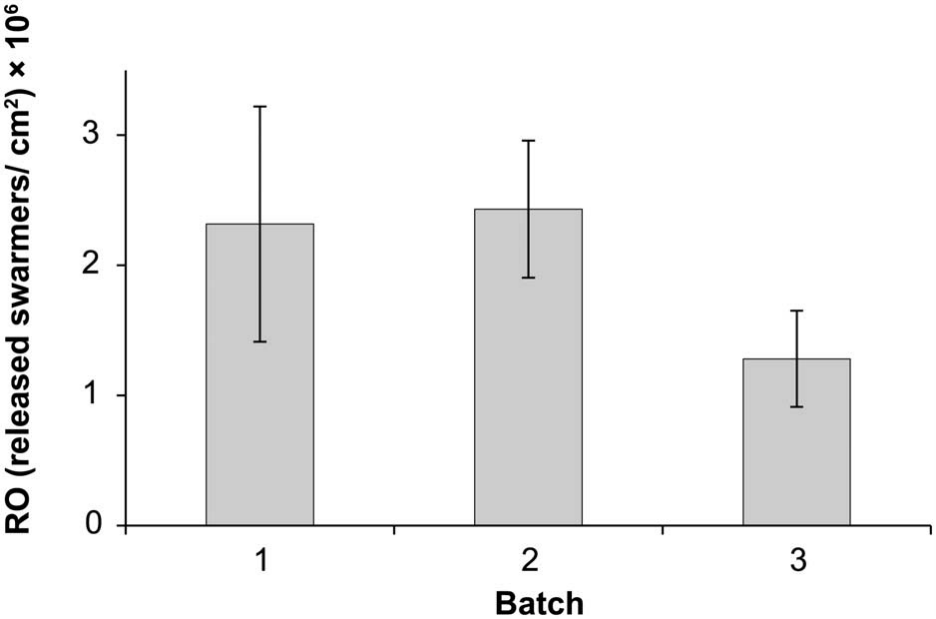
Reproductive output of *Ulva* sp. 3. Mean (± S.E.) reproductive output (RO) of three independently collected algal batches (*n = *50). Experiments were initiated on 6 May 2013 (Batch 1), 7 May 2013 (Batch 2), and 14 May 2013 (Batch 3).

The reproductive output differed between batches and was nearly doubled for batch 1 and 2 in comparison to batch 3 with 2.3±0.9, 2.4±0.5 and 1.3±0.4×10^6^ released swarmers per cm^2^, respectively (Figure 8). Biflagellate swarmers were much more common (95%; 56 out of 59 analysed samples) than quadriflagellate swarmers (5%; 3 out of 59 analysed samples).

Out of 150 thalli used to determine the viability and germling development of swarmers, a total of 147 thalli released swarmers. The released swarmers, both bi- and quadriflagellate, settled and germinated successfully in all 147 samples after five days.

#### Phototactic behaviour of released swarmers

A total of 22 thalli released swarmers, all of which were biflagellate with a negative phototactic response. However, on one occasion, a small number of released biflagellate swarmers showed positive phototaxis, while the vast majority of swamers released from the same thallus were negatively phototactic. The average length and width of the biflagellate swarmers was 6.55±0.85 (mean ± S.D.) and 3.75±0.52 µm, respectively.

## Discussion

**Figure 9:**
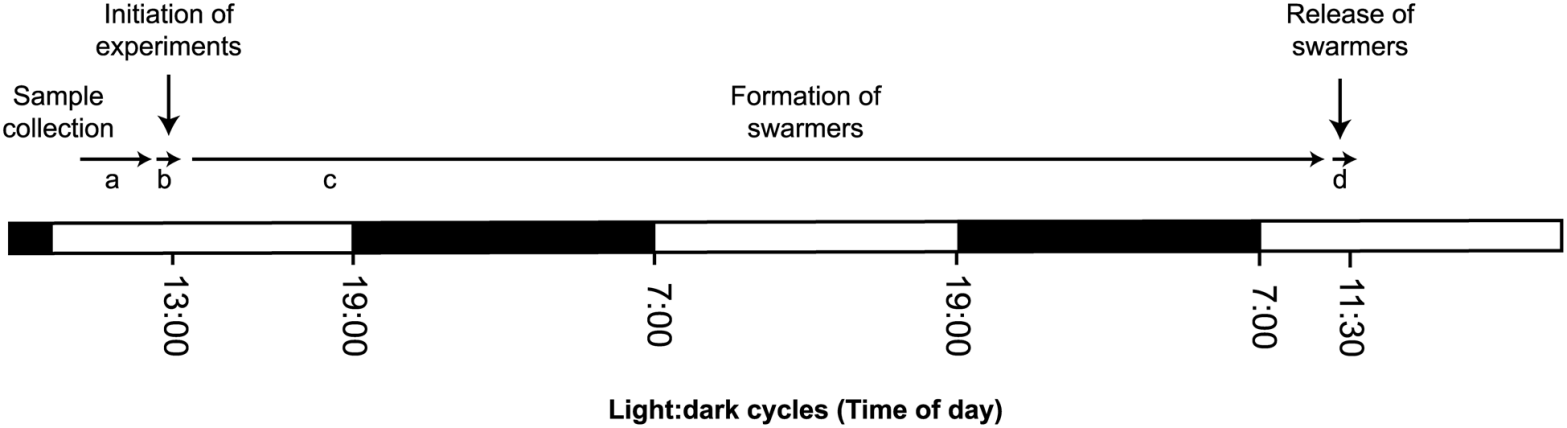
Time course of induced sporulation of tropical *Ulva sp 3*. (a) Collection of algal samples and subsequent transportation to the laboratory. (b) Initiation of experiments at 1 pm by washing the thalli in FSW, subsequently chill *Ulva* for 10 min and then place thalli into autoclaved FSW under a 12 h light:12 h dark photoeriod at 25°C. (c) Induction of sporulation with visible formation of swarmers after approximately 26 h. (d) Release of swarmers peaks between 10∶00 and 11∶30 am.

This study provides a foundation for understanding the factors affecting the reproduction of the filamentous species of tropical *Ulva*, and identifies the best treatment combination to induce reproduction of a common and widely distributed species under controlled conditions. Photoperiod and temperature shock were successfully manipulated to enhance the formation and release of swarmers, while the effects of segmentation, dehydration, salinity, and time of initiation of experiments were negligible. The efficient manipulation of photoperiod and temperature shock is therefore the key in the reliable supply of swarmers with applications for fouling studies of tropical *Ulva* species and the seeding of nets for mass-cultivation. The proposed method–factoring in the maximum release and minimum practical effort and timing–is therefore to collect *Ulva* in the morning and initiate the experiments in the early afternoon (at 1 pm) by washing the thalli in FSW, subsequently chilling the thalli for 10 min at 4°C and then placing them into autoclaved FSW under a 12 h L:12 h D photoperiod at 25°C. Consequently, swarmers are released with peak after two days between 10∶00 and 11∶30 am (Figure 9).

Notably, photoperiod had a significant effect on the formation and release of swarmers, with a discharge of up to 50% under normal photoperiod (12 h L:12 h D). In contrast, extended light periods resulted in lower discharge and this is in agreement with temperate environments, where the day length plays a key role in the reproduction of seaweed [22], [53]. While shorter days result in a reduced growth rate and trigger reproduction, longer days allow continuation of the vegetative growth phase for seaweeds in temperate systems [54]. However, unlike previous studies on *Ulva* in temperate and cold waters where continuous light and dark cycles restrained the discharge of swarmers [22], [55]–[57], tropical *Ulva* sp. 3 continued to form and release swarmers under both extremes (up to 23% and 12%, respectively).

The present study highlights the importance of the dark phase for the formation and release of swarmers in tropical *Ulva* sp. 3, as discharge was halved when thalli were kept under continuous light. In general, the dark phase is essential for the formation of swarmers as most cells of *Ulva* divide during the dark period [29], [58], [59], forming zoospores and gametes by meiosis and mitotic division, respectively [29], [35]. A dark phase of at least 1 h is essential to induce the release of swarmers of temperate *U. pseudocurvata* [22]. Furthermore, the dark period is an important factor to trigger the release of swarmers [22], with a discharge in the morning [26] after a few minutes [22] and up to 2–5 h after the onset of the light period [31] for *U. pseudocurvata* and *U. pertusa*, respectively. For *Ulva* sp. 3, the release of swarmers peaked at 4½ h after onset of light, around 11∶30 am.

A further factor affecting the formation and release of swarmers of *Ulva* sp. 3 was temperature shock. Sporulation was increased by approximately 10% when thalli were exposed to chilled (4°C) seawater for 10 min. This is consistent with a study in temperate waters identifying temperature shock as a trigger to induce reproduction of *U. lactuta* [36]. Furthermore, refrigeration of fertile thalli is also commonly used to maximise the release of swarmers in temperate environments [19], [48], [60]. The increased formation and release of swarmers following temperature shock may be a strategy to disperse under unfavourable conditions.

In general, stress treatments of segmentation [12], [61], dehydration [27], [39], [40], [62], and salinity [12] can initiate the formation and release of swarmers of *Ulva* [63]. In contrast, these factors had no effect on the sporulation of *Ulva* sp. 3. This was unexpected, particularly that segmentation did not result in an increase of sporulation as shown for *U. mutabilis*, where sporulation increased by more than 60% when thalli were fragmented [64]. Notably, the ‘punching method’ and other segmentation techniques are commonly used to induce reproduction of a wide range of *Ulva* species within a few days, including *U. fenestrata* [65], *U. ohnoi* [66], *U. pertusa* [31], [50], [56], [61], *U. prolifera* [12], [32], [33], *U. pseudocurvata* [22], *U. spinulosa* [21], and *U. mutabilis* [29], [59]. It is unclear at this stage whether the traits listed above are uniquely temperature or sub-tropical and do not have direct relevance to tropical species.

The dehydration of filaments was also ineffective in increasing the sporulation of *Ulva* sp. 3. Previous studies have used this method to maximise the release of swarmers and dehydration times ranged from less than 1 h [27] up to 12 h [39], [40]. Therefore, the tested dehydration times of 45 min and 4 h are well within these timeframes and provide confidence that dehydration is ineffective for tropical *Ulva* sp. 3. However, dehydration was proven to be effective for other tropical *Ulva* species [37]–[40]. In addition, neither dehydration nor wrapping in moist paper towel were effective constraining treatments for *Ulva* sp. 3 in the present study and swarmers were released during the desiccation process, indicating that once the reproductive cells are ready for release, this process might not be delayed.

The overall discharge of swarmers was generally low, at approximately 50% of previous studies, where sporulation reached up to 90–100% [12], [31], [37], [64]. In general, the discharge differs between sporophytes and gametophytes, with more than 90% in sporophytes, and only 40% for gametophytes [31]. A total of 95% of thalli in the present study released biflagellate swarmers. The possession of a majority of negative phototaxis and the ability to germinate and grow within five days without fusing with complementary gametes suggests a simple asexual life history via biflagellate zoids [17]. However, this requires confirmation through the cultivation of successive generations. In general, gametes are positively phototactic [34], [67], [68], while asexual biflagellate zoids are negatively phototactic [17], as are quadriflagellate zoospores [37] and zygotes [34]. The negative phototaxis guides swarmers to suitable surfaces for settlement and attachment [69]. Quadriflagellate swarmers were also released in the present study, although only from 5% of the thalli, and it remains unknown as to whether these are zoospores or asexual zoids [21]. In general, species of *Ulva* with a simple asexual life history produce either exclusively bi- of quadriflagellate zoids [17], [21]. It is possible that both bi- and quadriflagellate swarmers were found in this study because used samples could have been from different species of *Ulva* due to morphological similarity between filamentous species of *Ulva*; alternatively *Ulva* sp. 3 may have several life histories as reported for *U. prolifera* (formerly *Enteromorpha prolifera*; [17]). Overall, the simplicity of the reproduction of *Ulva* sp. 3 may be a unique trait of tropical species related to *Ulva* sp. 3 that has genetic basis. Based on phylogenetic trees constructed using ITS sequences, *Ulva* sp. 3 forms a unique clade separate to other species that have been investigated in previous studies.

In conclusion, a baseline method to induce the sporulation in tropical *Ulva* sp. 3 within 30 to 48 h after initiation was established. Sporulation was enhanced by temperature shocking thalli prior to incubating at a photoperiod of 12 h L:12 h D. Swarmers were released two days after initiating experiments with a maximum release between 10∶00 and 11∶30 am. A total of 95% of the collected field population released biflagellate swarmers with negative phototaxis. These swarmers also had the ability to settle and germinate without crossing with complementary gametes. The findings of this study have application in laboratory antifouling assays for the tropics and in the seeding of nets for the mass-cultivation of seaweed.

## Supporting Information

Figure S1 ***Ulva*** **ITS phylogenetic tree.** Maximum likelihood tree of *Ulva* internal transcribed spacer (ITS) sequence data (scale at bottom). Numbers near each node refer to bootstrap support values, nodes with <50% bootstrap support are not labelled. Sample used in this study shown in bold. Numbers accompanying the species names are GenBank accession numbers for the sequences used in the analysis.
(TIF)

Text S1 (DOCX)
