# Supplementary figures and images for: Methods for the Induction of Reproduction in a Tropical Species of Filamentous Ulva
Source: PLoS One. 2014 May 13;9(5):e97396. doi: 10.1371/journal.pone.0097396 (PMC4019596; doi:10.1371/journal.pone.0097396)

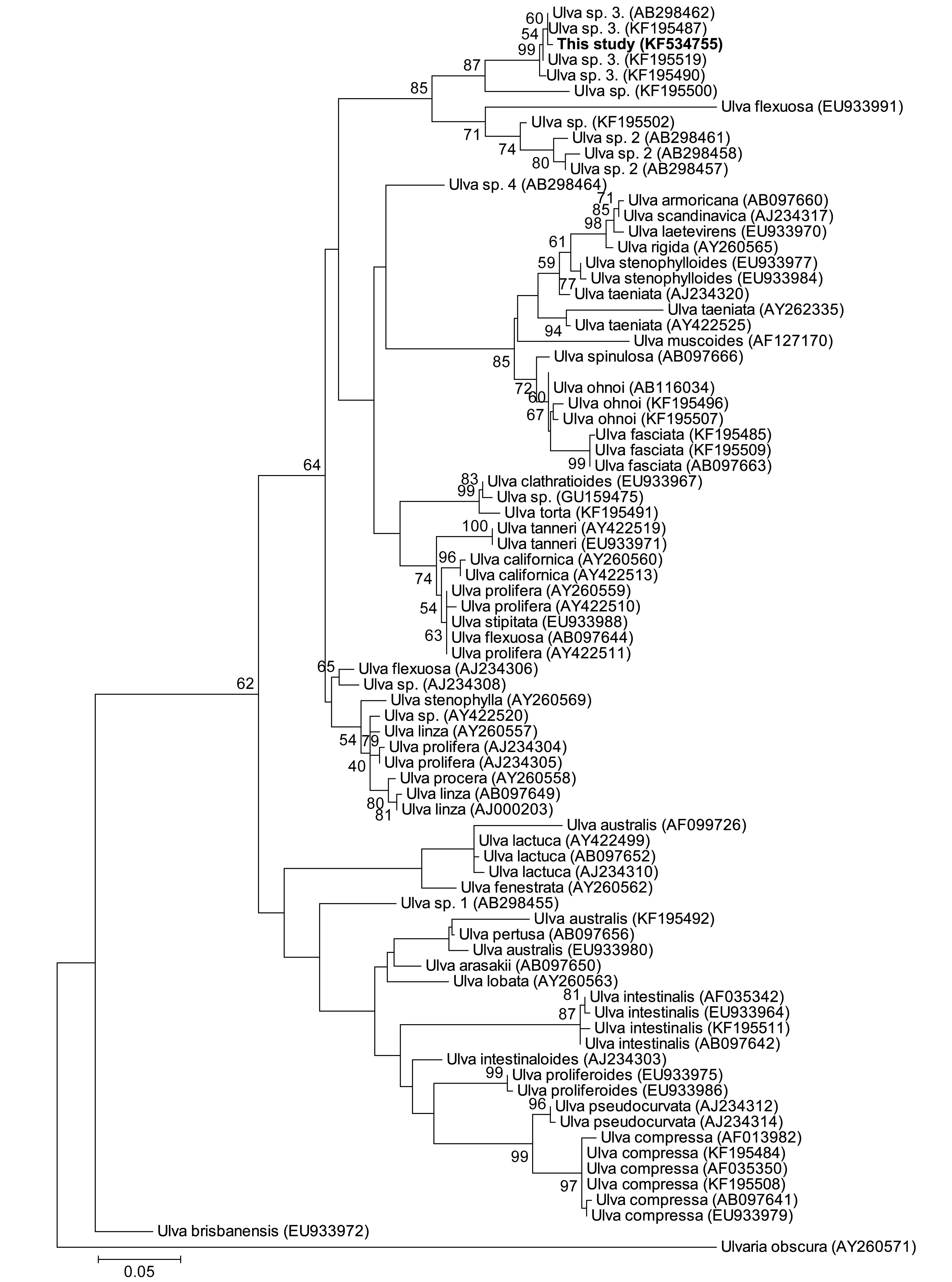

Supplement: Figure S1 — Ulva ITS phylogenetic tree. Maximum likelihood tree of Ulva internal transcribed spacer (ITS) sequence data (scale at bottom). Numbers near each node refer to bootstrap support values, nodes with <50% bootstrap support are not labelled. Sample used in this study shown in bold. Numbers accompanying the species names are GenBank accession numbers for the sequences used in the analysis. (TIF) [file pone.0097396.s001.tif]
