# Supplementary material for: Methods for the Induction of Reproduction in a Tropical Species of Filamentous Ulva
Source: PLoS One. 2014 May 13;9(5):e97396. doi: 10.1371/journal.pone.0097396 (PMC4019596; doi:10.1371/journal.pone.0097396)
Supplement: Text S1 — (DOCX) [file pone.0097396.s002.docx]

**SUPPORTING INFORMATION**

**Text S1**

***DNA barcoding***

Total DNA was extracted from two dried samples of *Ulva* using a Qiagen DNEasy Plant Mini Kit following the manufacturer’s instructions and amplified at the DNA barcoding marker ITS using the primers ITS1 [1] and G4 [2]. Polymerase chain reaction (PCR) amplifications were performed in a 25 µL reaction mixture containing 1.5 U of MyTaq HS DNA polymerase (BiolineTM), 5 x MyTaq reaction buffer, 0.4 µM each primer, and 1 µL of genomic DNA (25 – 30 ng). Amplification was performed on a BioRad C1000 Thermal Cycler using a touchdown protocol (cycling parameters: 2 min at 94 °C, 30 cycles of 30 s denaturing at 94 °C, 45 s annealing at 48 - 56 °C, 60 s extension at 72 °C, and a final extension at 72 °C for 10 min). PCR products were column purified using Sephadex G-25 resin and sequenced in both directions by the Australian Genome Research Facility, Brisbane, Australia. Sequences were edited using Sequencher v4.5 (Gene Codes Corporation, Ann Arbor, MI, USA) and submitted to GenBank (Accession number KF434755). Sequences from the two samples of DNA were identical.

Sequence similarity searches using a nucleotide BLAST search in GenBank (<http://www.ncbi.nlm.nih.gov/BLAST/>) failed to find an exact match. Therefore we identified our sample using phylogenetic trees constructed with sequences downloaded from Genbank. We downloaded all newly generated sequences from the recent studies of Australian *Ulva* by Lawton et al. [3] and Kraft et al. [4], and of Hawaiian *Ulva* by O’Kelly et al. [5] as well as all previously published ITS *Ulva* sequences used in these studies for analysis. Duplicate sequences were removed from the dataset and then all remaining sequences were aligned with ours and trimmed to a standard length in MEGA 5.0 [6]. The dataset included 105 sequences and the alignment consisted of 595 positions. Maximum likelihood (ML) phylogenetic trees were constructed in MEGA using an *Ulvaria obscura* sequence (Genbank accession: AY260571) as an outgroup. jModelTest 2.1 [7, 8] showed that the TIM1+G model of molecular evolution best fitted the data. However, as this model was not available in MEGA we used the simple Kimura two-parameter model to estimate genetic distance [9] as this is the standard model of molecular evolution used in barcoding studies [10]. The reliability of tree topologies was estimated using bootstrapping (1,000 replicates). Our sample formed a distinct, well supported (99% bootstrap support) clade with a GenBank sample from Japan identified as *U. sp. 3* (AB298458, [11]) (Fig S1). Therefore we have identified our sample as *U. sp. 3* following Lawton et al. [3].

**References**

1. Bakker FT, Olsen JL, Stam WT (1995) Evolution of nuclear rDNA its sequences in the *Cladophora albida/sericea* clade (Chlorophyta). J Mol Evol 40: 640–651.

2. Harper J, Saunders G (2001) The application of sequences of the ribosomal cistron to the systematics and classification of the florideophyte red algae (Florideophyceae, Rhodophyta). Cah Biol Mar 42: 25–38.

3. Lawton RJ, Mata L, de Nys R, Paul N (2013) Algal bioremediation of waste waters from land-based aquaculture using *Ulva*: selecting target species and strains. PLoS One 8(10): e77344.

4. Kraft LGK, Kraft GT, Waller RF (2010) Investigations into southern Australian *Ulva* (Ulvophyceae, Chlorophyta) taxonomy and molecular phylogeny indicate both cosmopolitanism and endemic cryptic species. J Phycol 46: 1257–1277.

5. O’Kelly CJ, Kurihara A, Shipley TC, Sherwood AR (2010) Molecular assessment of *Ulva* spp.(Ulvophyceae, Chlorophyta) in the Hawaiian Islands. J Phycol 46: 728–735.

6. Tamura K, Peterson D, Peterson N, Stecher G, Nei M, et al. (2011) MEGA5: molecular evolutionary genetics analysis using maximum likelihood, evolutionary distance, and maximum parsimony methods. Mol Biol Evol 28: 2731–2739.

7. Guindon S, Gascuel O (2003) A simple, fast, and accurate algorithm to estimate large phylogenies by maximum likelihood. Syst Biol 52: 696–704.

8. Darriba D, Taboada GL, Doallo R, Posada D (2012) jModelTest 2: more models, new heuristics and parallel computing. Nat Meth 9: 772.

9. Kimura M (1980) A simple method for estimating evolutionary rates of base substitutions through comparative studies of nucleotide sequences. J Mol Evol 16: 111–120.

10. Hebert PDN, Ratnasingham S, de Waard JR (2003) Barcoding animal life: cytochrome c oxidase subunit 1 divergences among closely related species. Proc R Soc London Ser B Biol Sci 270: S96–S99.

11. Shimada S, Yokoyama N, Arai S, Hiraoka M (2008) Phylogeography of the genus *Ulva* (Ulvophyceae, Chlorophyta), with special reference to the Japanese freshwater and brackish taxa. J Appl Phycol 20: 979–989.
